# m6A‐related lncRNAs as potential biomarkers and the lncRNA ELFN1‐AS1/miR‐182‐5p/BCL‐2 regulatory axis in diffuse large B‐cell lymphoma

**DOI:** 10.1111/jcmm.18046

**Published:** 2023-12-01

**Authors:** Qinglong Yang, Yingxue Lu, Ashuai Du

**Affiliations:** ^1^ Department of General Surgery Guizhou Provincial people's Hospital Guiyang China; ^2^ Department of Infectious Diseases Guizhou Provincial people's Hospital Guiyang China

**Keywords:** ABT‐263, BCL‐2, DLBCL, ELFN1‐AS1, lncRNA, M6A, miR‐182‐5p

## Abstract

Diffuse large B‐cell lymphoma (DLBCL) is the most common lymphoid subtype. However, unsatisfactory survival outcomes remain a major challenge, and the underlying mechanisms are poorly understood. N6‐methyladenosine (m6A), the most common internal modification of eukaryotic mRNA, participates in cancer pathogenesis. In this study, m6A‐associated long non‐coding RNAs (lncRNA) were retrieved from publicly available databases. Univariate, LASSO, and multivariate Cox regression analyses were performed to establish an m6A‐associated lncRNA model specific to DLBCL. Kaplan–Meier curves, principal component analysis, functional enrichment analyses and nomographs were used to study the risk model. The underlying clinicopathological characteristics and drug sensitivity predictions against the model were identified. Risk modelling based on the three m6A‐associated lncRNAs was an independent prognostic factor. By regrouping patients using our model‐based method, we could differentiate patients more accurately for their response to immunotherapy. In addition, prospective compounds that can target DLBCL subtypes have been identified. The m6A‐associated lncRNA risk‐scoring model developed herein holds implications for DLBCL prognosis and clinical response prediction to immunotherapy. In addition, we used bioinformatic tools to identify and verify the ceRNA of the m6A‐associated lncRNA ELFN1‐AS1/miR‐182‐5p/BCL‐2 regulatory axis. ELFN1‐AS1 was highly expressed in DLBCL and DLBCL cell lines. ELFN1‐AS1 inhibition significantly reduced the proliferation of DLBCL cells and promoted apoptosis. ABT‐263 inhibits proliferation and promotes apoptosis in DLBCL cells. In vitro and in vivo studies have shown that ABT‐263 combined with si‐ELFN1‐AS1 can inhibit DLBCL progression.

## 1 INTRODUCTION

Diffuse large B‐cell lymphoma (DLBCL) is the most common lymphoid malignancy among adults, which is characterized by heterogeneous phenotypes and can transform from more inert lymphoma types,^1^ such as follicular lymphoma and chronic lymphocytic leukaemia.^2^ Although persistent mitigation is realized in more than 50% of patients, even in the advanced stage of the disease, DLBCL remains a daunting clinical challenge, with one in three patients not being cured with conventional treatments such as immunochemotherapy.^3^

N6‐methyladenosine (m6A) is the most common epigenetic modification of mRNA and long non‐coding RNA (lncRNAs) and is vital for RNA splicing, export, stability and translation.^4^ m6A modification is a reversible, dynamic RNA epigenetic process modulated by m6A modulators, like ‘writers’ (methyltransferases), ‘readers’ (signal transducers) and ‘erasers’ (demethylases).^5^ Furthermore, the m6A modification is an invertible RNA epigenetic process.^6^ Changes in RNA levels can influence various cellular processes; hence, m6A‐modulated lncRNAs may be critical for cancer cell growth and metastasis.^7^

m6A modifications regulate tumourigenesis and tumour progression. For example, METTL3, affected by m6A modifications, regulates the METTL3/PEDF axis and promotes DLBCL cell proliferation.^8^ piRNA‐30473 regulates RNA m6A methylation in DLBCL via the piRNA‐30473/WTAP/HK2 axis, thereby promoting tumourigenesis and resulting in a poor prognosis.^9^ Recently, it has been shown that the aberrant regulation of m6A modulators is involved in DLBCL.^8^, ^10^ The specific function of m6A modulators in lncRNAs remains elusive, revealing that the causal links between m6A‐associated lncRNAs and DLBCL progression may facilitate the discovery of prognosis‐related targets.

In this study, we extracted the expression profiles of 1103 lncRNAs and 23 m6A genes from the Gene Expression Omnibus (GEO), The Cancer Genome Atlas (TCGA) and the Genotype‐Tissue Expression (GTEx) databases. Data from the GEO dataset, GSE10846, were used for a more in‐depth analysis. Next, we used Pearson's correlation analysis to identify the lncRNAs associated with m6A. A model based on these lncRNAs was developed to predict the overall survival (OS) of patients with DLBCL. Using an open‐access drug sensitivity database, compounds that target m6A‐associated lncRNA hallmarks were identified, and their association with immune therapy responses was determined. Finally, we investigated the expression profiles of the m6A‐associated lncRNAs and verified their potential regulatory mechanisms in DLBCL.

## 2 MATERIALS AND METHODS

### 2.1 Data collection

Using the VarScan program, we acquired RNA sequencing transcriptome‐associated clinical and variant data for patients with DLBCL from the GEO, TCGA and GTEx databases. The study process is illustrated in Figure S1.

### 2.2 Selection of m6A genes and m6A‐associated lncRNAs

Data on lncRNAs and m6A were retrieved from the aforementioned databases. We acquired expression profiles of 23 m6A modifications: *METTL3*, *METTL14*, *METTL16*, *VIRMA*, *RBM15*, *RBM15B*, *ZC3H13* and *WTAP*; the readers *IGF2BP1*, *IGF2BP2*, *IGF2BP3*, *YTHDC1*, *YTHDC2, YTHDF1*, *YTHDF2*, *YTHDF3*, *HNRNPA2B1*, *HNRNPC*, *LRPPRC*, *RBMX, FMR1* and erasers *ALKBH5* and *FTO*. Pearson's correlation analysis identified 293 m6A‐associated lncRNAs. The inclusion criteria were |Pearson's R| > 0.4 and *p* < 0.001.

### 2.3 Construction and verification of the risk signature

We first integrated the data from the TCGA and GEO databases, performed batch correction to reduce variance and obtained a joint dataset. The TCGA and GEO datasets were randomly divided into learning and testing sets. The learning set was used to construct the m6A‐associated lncRNA model. TCGA and GEO datasets and testing sets were used to verify the constructed model. No remarkable differences were observed in the clinical performance between the two datasets (*p* > 0.05). Combining the survival information of patients with DLBCL from TCGA and GEO, we selected the prognostic results of 293 m6A‐associated lncRNAs from the TCGA and GEO datasets (*p* < 0.05) and performed univariate Cox regression analysis.^11^ Using the glmnet package in R, which is used for LASSO Cox regression analysis (via penalized parameters speculated by 10‐fold cross‐verification),^12^ 25 m6A‐associated lncRNAs were observed to be significantly associated with OS in patients with DLBCL in the GEO and TCGA datasets. After subjecting these 25 m6A‐associated lncRNAs to a multivariate Cox regression analysis, three m6A‐associated lncRNA risk models were developed. Subgroups, including low‐ and high‐risk groups, were established according to the mid‐value of the risk scoring.^13^

### 2.4 Function analysis

We performed gene ontology (GO) enrichment analyses to determine the differentially expressed genes using the package clusterProfiler in R. *p* < 0.05 indicated significant enrichment of the functional annotations.^14^, ^15^

### 2.5 Responses to immunotherapy

The R package maftools were used to assess the variant data. Tumour mutational burden (TMB) has been identified as a cancer‐specific genetic mutations.^16^, ^17^

### 2.6 Principal component and Kaplan–Meier survival analyses

Principal component analysis (PCA) was utilized for efficient dimension reduction, model recognition and group visualization of high‐dimensional data, including whole‐genome expression profiles,^18^ Using Kaplan–Meier (K–M) survival analyses to evaluate the variation in the OS between the low‐ and high‐risk groups. The R packages survMiner and survival were used for this purpose.^19^

### 2.7 Identification of compounds targeting m6A‐associated lncRNAs

To identify potential drug candidates for the treatment of DLBCL, we used the R package pRRophetic and determined the half‐maximal inhibitory concentration (IC50) of compounds, for which data were acquired from the Genomics of Drug Sensitivity in Cancer (GDSC).

### 2.8 Establishing a prognostic model

The predictive power of independent factors (age, sex and risk scoring) for 1‐, 3‐ and 5‐year OS was estimated. Calibration curves based on the Hosmer–Lemeshow assay were used to describe the association between the actual and model‐predicted outcomes.

### 2.9 RNA extraction and reverse transcription‐quantitative PCR (RT‐qPCR)

Plasma samples were collected from 60 patients with DLBCL and 60 normal controls (NC) at Guizhou Provincial People's Hospital. This study was approved by the Ethics Committee of our hospital. Total RNA from lineage cells and DLBCL and NC plasma samples was prepared using the TRIzol reagent (Invitrogen). Reverse transcription was performed using the RevertAid First‐Strand cDNA Prep Tool (Thermo Fisher Scientific). Gene expression was normalized to *GAPDH* expression. Faststart Universal SYBR Green Master Mix (Roche) was used for qPCR assays on a StepOne thermal cycler (Applied Biosystems). Relative fold changes in expression were analysed via the 2^−ΔΔCT^ approach.

The primer sequences used in our study were as follows:

ELFN1‐AS1 forward 5′‐TAGGAATGTGGCGGATGGTGA‐3′ and reverse 5′‐GGAAGCGTGTAGGAAGCGTGG‐3′.

BCL‐2 forward 5′‐CGAGTGGGATGCGGGAGATG‐3′ and reverse 5′‐CGGGATGCGGCTGGATGG‐3′.

GAPDH forward 5′‐GGACGCATTGGTCGTCTGG‐3′ and reverse 5′‐TTTGCACTGGTACGTGTTGAT‐3′.

### 2.10 Cell culture and treatment

Diffuse large B‐cell lymphoma cell lines (TMD8, OCl‐LY8, HBL1 and SU‐DHL‐6) and B cells were obtained from the ATCC (ATCC). DLBCL cells were cultured in RPMI‐1640 medium (Hyclone; GE Healthcare) and stored in a humidified incubator at 37°C, 5% CO2. When the cells reached 50% confluence, they were treated with DMSO or various concentrations of ABT‐263 (Selleck Chemicals).

### 2.11 Cell proliferation

Diffuse large B‐cell lymphoma cells were cultured in 96‐well plates (2 × 10^5^ cells/well). After incubation at 37°C and 5% CO2 for varying durations, 10 μL of CCK‐8 (Dojindo) was added and maintained for an additional 4 h. A microplate reader (Potenov) was used to measure the absorbance at 450 nm.

### 2.12 Cell apoptosis assay

Apoptosis was measured using an Apoptosis Detection Kit (Sigma). DLBCL cells (2 × 10^5^ cells per well) were seeded in 12‐well plates. After 24 h of treatment, the cells were collected and treated with Annexin V‐binding buffer, then labelled with Annexin V‐FITC and PI (Sigma). The percentage of apoptotic cells was assessed using flow cytometry.

### 2.13 Dual‐luciferase assay

The online tool TargetScan was used to identify the potential binding sites. Wild‐type (wt) and mutant site (mut) sequences of ELFN1‐AS1 (ELFN1‐AS1 wt and ELFN1‐AS1 mut) and BCL‐2 (BCL‐2 wt and BCL‐2 mut), including the homologous binding sites of miR‐185‐5p, were amplified and uniformly plugged into the vector pGL3 (Promega). A dual‐luciferase reporter assay system (Promega) was used to detect luciferase activity.

### 2.14 Tumour transplantation in NOD/SCID mice

NOD/SCID mice were fed with a specific pathogen in an animal laboratory. The mice were randomly divided into four groups, with six per group. A cell suspension (0.1 mL; 1 × 10^7^) was prepared from SU‐DHL6 cells from different treatments and injected subcutaneously into the neck and back. When the tumour volume reached approximately 50 mm^3^, the animals were randomly divided into four groups: PBS, si‐ELFN1‐AS1; ABT‐263, and si‐ELFN1‐AS1 + ABT‐263 (*n* = 6 mice per group) and treated with different formulations of si‐ELFN1‐AS1 (20 mg siRNA per mouse equivalent) and ABT‐263 (75 mg/kg per mouse equivalent) via intraperitoneal vein injection once a week. On Day 28, all animals were slaughtered, and the following formula was used to quantify tumour volume: V (volume) = (length width2)/2. The tumour tissue was extracted and imaged. The tumours were then extracted for histopathological analysis.

### 2.15 Haematoxylin and eosin

Using a microtome, 4 μm sections were obtained from each paraffin block. The sections were immersed in xylene for 10 min, rehydrated with absolute ethanol (95%, 85% and 70% ethanol) for 5 min, immersed and washed thrice with PBS (phosphate buffered solution) for three times, 5 min each. Then, 100 μL of pre‐prepared haematoxylin solution was added to each tissue section and stained for 10 min. The sections were stained with an eosin solution for 3 min, dehydrated with graded alcohol and cleared in xylene. Finally, slides were mounted using a neutral resin.

### 2.16 Immunohistochemistry

The tissues from NOD/SCID mice were cut into 4 μm slides. The antibodies against Ki‐67, Bax and BCL‐2 were purchased from Cell Signalling Technology. Immunohistochemistry analysis was performed as previously described. Images were obtained under a microscope (Olympus) at appropriate magnification.

### 2.17 Statistical analysis

One‐way analysis of variance (anova) and paired sample *t*‐tests were used to assess differences between groups. Pearson's correlation test was used to analyse the correlations. SPSS 23.0 software and GraphPad Prism 7.0.1 were performed for statistical analyses. All experiments were performed independently and repeated thrice. *p* < 0.05 was considered statistically significant.

## 3 RESULTS

### 3.1 Identification of m6A‐associated lncRNAs in DLBCL

**FIGURE 1:**
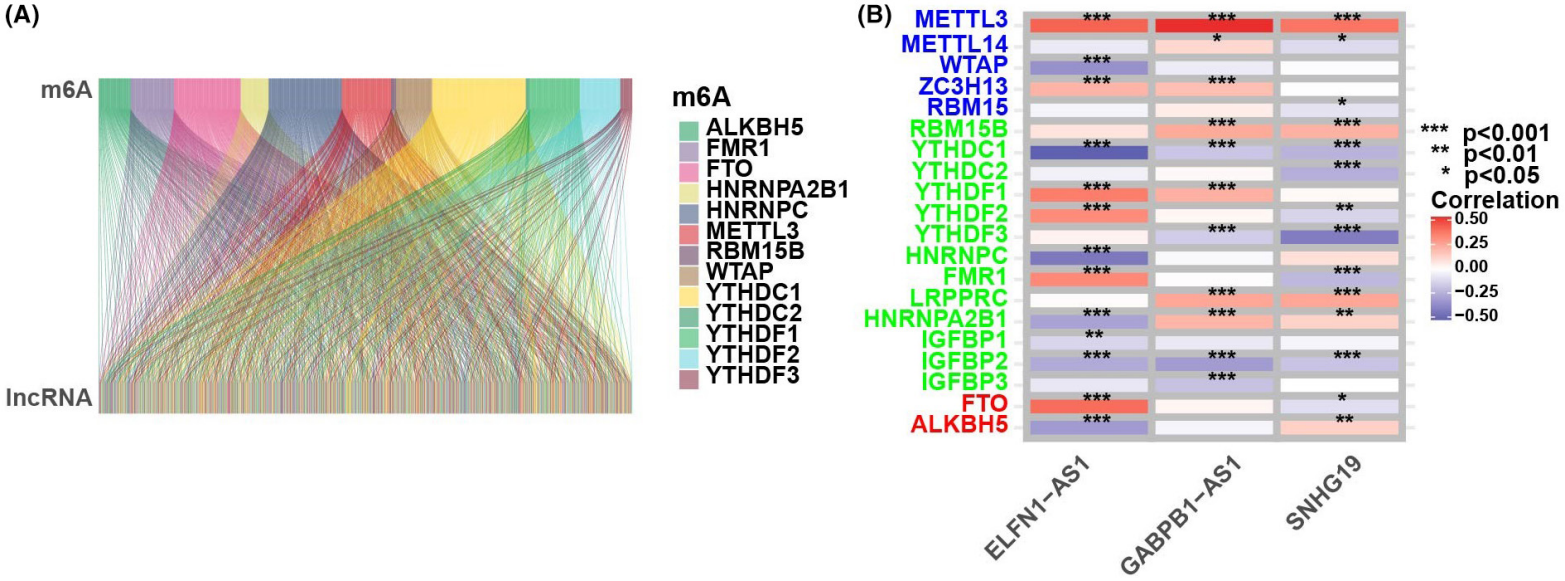
Determination of m6A‐associated lncRNAs in DLBCL sufferers. (A) Sankey relation chart for 23 m6A genes and m6A‐associated lncRNAs. (B) Heat map for the association between 23 m6A genes and the three prognostic m6A‐associated lncRNAs.

The expression profiles of 23 m6A genes and 1103 lncRNAs were extracted from TCGA, GTEx and GEO databases. We defined m6A‐associated lncRNAs as those that were significantly associated with one or more of the 23 m6A genes (|Pearson's R| > 0.4 and *p* < 0.001). Figure 1A describes a Sankey plot of the m6A‐lncRNA co‐expression network; 293 m6A‐associated lncRNAs were identified. The association between m6A‐related genes and m6A‐associated lncRNAs in the GEO and TCGA datasets is shown in Figure 1B.

### 3.2 Establishment and verification of a risk model based on m6a‐Associated lncRNAs in DLBCL

**FIGURE 2:**
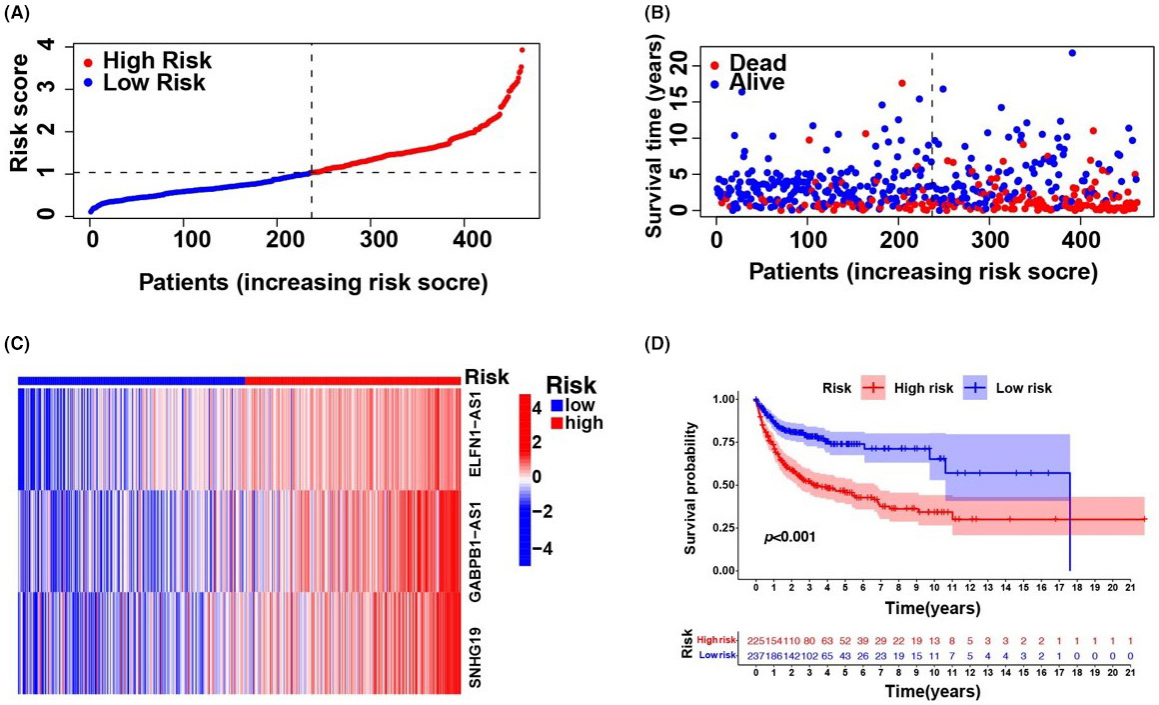
Prognosis significance of the risk features of the three m6A‐associated lncRNAs in the entire TCGA and GEO set. (A) Distributional status of m6A‐associated lncRNA model risk scoring. (B) Diverse survival duration and status features exist between the low‐risk and high‐risk groups. (C) Heat map of cluster analyses describes the expression criteria of the three prognostic lncRNAs for every sufferer. (D) K–M curves of the patient OS in the low‐risk group and high‐risk group.

Univariate Cox regression analysis was used to select m6A‐associated lncRNAs (from 293 m6A‐associated lncRNAs in the training set comprising data from all three databases) that could be helpful for DLBCL prognosis. We observed that 25 m6A‐associated lncRNAs in the TCGA and GEO databases were significantly associated with OS (Figure S2A). LASSO‐penalized Cox analyses are common multi‐regression analyses, the utilization of which improves the predictive accuracy and interpretability of statistical models and enables simultaneous variate selection and regularization. This approach is widely used for the optimal selection of features with low correlations and prominent predictive values in high‐dimensional data to avoid overfitting. The approach can, therefore, help validate the most predictive biomarkers and generate prognostic indices for determining clinical outcomes. The dotted line describes the first rank value of log λ with minimal segment likelihood bias. Therefore, 25 m6A‐associated lncRNAs were chosen for the following multivariable analyses (Figure S2B,C). DLBCL specimens were categorized into low‐ and high‐risk groups based on mid‐value risk scoring. The distribution of risk scores between the groups is described in Figure 2A, and the survival duration and status of patients in these groups are described in Figure 2B. The comparative expression criteria for the three m6A‐associated lncRNAs in all patients are described in Figure 2C. Survival analyses showed that the OS of the low‐risk group was better than that of the high‐risk group (*p* < 0.001; Figure 2D).

**FIGURE 3:**
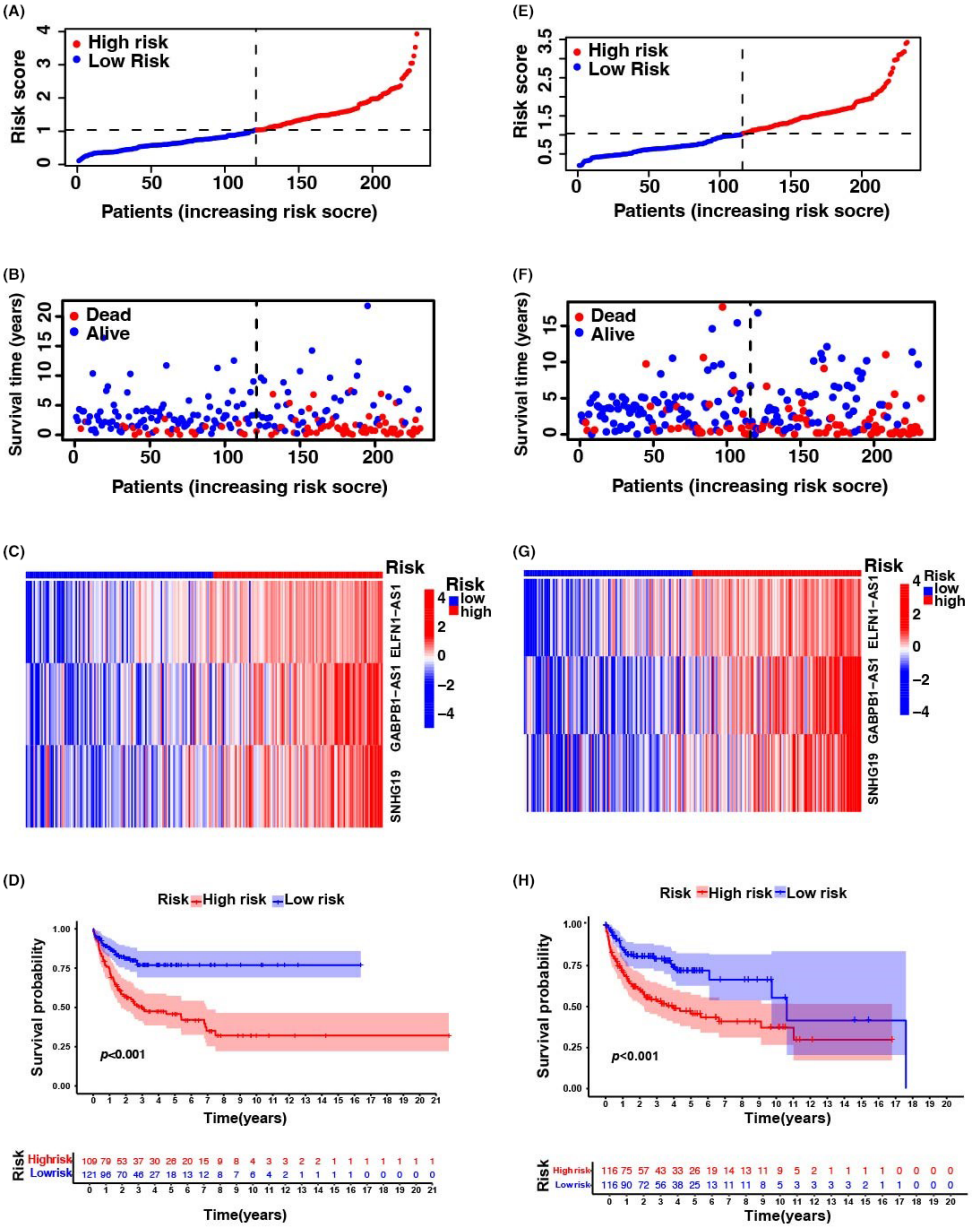
Prognosis significance of the risk modelling of the three m6A‐associated lncRNAs in the test set and learning set. (A) Distributional status of m6A‐associated lncRNA model risk scoring for the test set. (B) Features of the survival status and duration between the low‐risk and high‐risk groups for the test set. (C) Heat map of cluster analyses describes the contents of the three prognostic lncRNAs for every sufferer within the test set. (D) K–M curves of the patient OS in the low‐risk and high‐risk groups for the test set. (E) Distributional status of the m6A‐associated lncRNA model risk scoring for the learning set. (F) Features of the survival duration and status between the low‐risk and high‐risk groups for the learning set. (G) Heat map of cluster analyses presents the expressing levels of the three prognostic lncRNAs for every sufferer in the learning set. (H) K–M curves of patient OS within the low‐risk and high‐risk groups for the learning set.

To test the prognostic utility of the developed model, we computed the risk scoring for all patients within the testing and complete sets of TCGA and GEO using a universal formula. Figure 3 describes the distribution status of risk scoring, survival duration and status features and the expression of m6A‐associated lncRNAs in the testing described in (Figure 3A–C) and training (Figure 3E–G) sets. K–M survival analysis of the testing and learning sets revealed differences in the TCGA learning set; the OS of DLBCL patients with a higher risk score was poorer than that of patients with a lower risk score (Figure 3D–H). Differences in OS stratified by clinicopathological features were analysed between the low‐ and high‐risk groups in the TCGA and GEO datasets. The subsets were categorized according to age (≤65 and >65 years) and sex (female and male). The OS of the low‐risk group was better than that of the high‐risk group when stratified according to age (*p* < 0.001) and sex (*p* < 0.001 for women; *p* = 0.008 for men) (Figure S3A–D).

### 3.3 PCA confirms the grouping capability of the m6A‐associated lncRNA model

Principal component analysis was conducted to examine the differences between the low‐ and high‐risk groups based on whole‐genome expression profiles, 23 m6A genes, 293 m6A‐associated lncRNAs and a risk model delineated by the expression profiles of three m6A‐associated lncRNAs (Figure S4A–D). The distributions of the low‐ and high‐risk groups were comparatively dispersed (Figure S4A–C). Nevertheless, the outcomes obtained using our model demonstrated that the low‐ and high‐risk groups had diverse distributions (Figure S4D). These results suggested that the prognostic characteristics differed between the two groups.

### 3.4 Tumour immune microenvironment and tumour immunotherapy response

**FIGURE 4:**
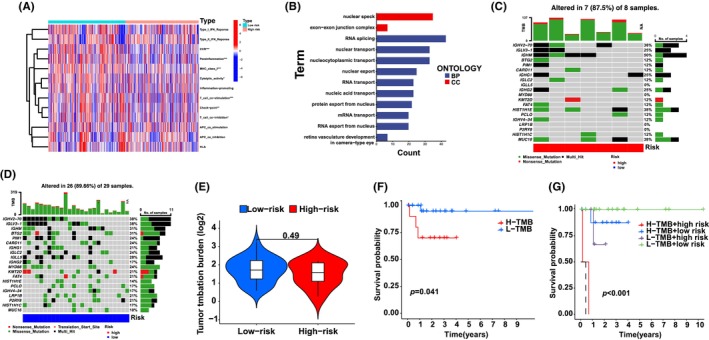
Speculation of the TIM and tumour immune therapy reactions via m6A‐associated lncRNA modelling in the entire TCGA and GEO set. (A) The indicated criteria of the immune indicator for every sufferer. (B) GO enrichment analyses. (C and D) The water fall plot describes variant data for the genes with great variant frequencies in the high‐risk group (C) and low‐risk group (D). (E) TMB diversity within the low‐risk and high‐risk groups. (F) Survival analysis for low TMB group and high TMB group via K–M curves (*p* = 0.041, log‐rank test). (G) Survival analysis for sufferers categorized by high‐risk group and TMB via K–M curves. H = high; L = Low (*p* < 0.001, log‐rank test).

The enrichment status and activity of specific immunocytes and pathways in DLBCL were studied using an m6A‐associated lncRNA model and 462 DLBCL samples. No differences were observed in the expression of immune indicators between low‐ and high‐risk groups (Figure 4A).

Our team completed GO enrichment analyses to explore potential molecular‐level causal links based on m6A modelling and observed that multiple immunity‐associated bioprocesses were involved (Figure 4B). Subsequently, we explored the correlation between the m6A‐associated lncRNA model and immunotherapy markers. The variant data were summarized using the R package maftools and stratified according to predictive factors and mutation effects. The top 20 genes with the highest frequency of mutations in the low‐ and high‐risk groups are described in Figure 4C,D. The top five mutated genes were *IGHV2‐70*, *IGLV3‐1*, *IGHM*, *BTG2* and *PIM1*. Next, TMB was assessed using TCGA somatic mutation data. No differences were observed in TMB levels between the low‐ and high‐risk groups, indicating that the m6A‐based classifier index did not correlate with TMB (*p* = 0.49; Figure 4E). We determined the prognostic value of low‐ and high‐risk TMB. The patients were categorized into low‐ and high‐risk groups. A significant survival advantage was observed in the low‐risk group (*p* = 0.041; Figure 4F), whereas patients in the low‐ and high‐risk groups showed a more remarkable survival advantage (*p* < 0.001; Figure 4G). These results suggest that low‐ or high‐risk factors can be used to evaluate the clinical prognosis of patients with DLBCL.

### 3.5 Prospective compounds that target m6A‐associated lncRNAs

To identify drugs or compounds that target m6A‐associated lncRNAs useful in DLBCL therapy, we determined the treatment reaction based on the IC50 of each compound deposited in the GDSC database. We identified 138 compounds, of which nine showed remarkable differences in the estimated IC50 between the risk groups; the high‐risk group was more sensitive to five compounds. Figure S5 shows the top nine compounds that may be utilized for further analyses of DLBCL treatment.

### 3.6 Assessment of the m6A‐associated lncRNA model and clinical characteristics of DLBCL

**FIGURE 5:**
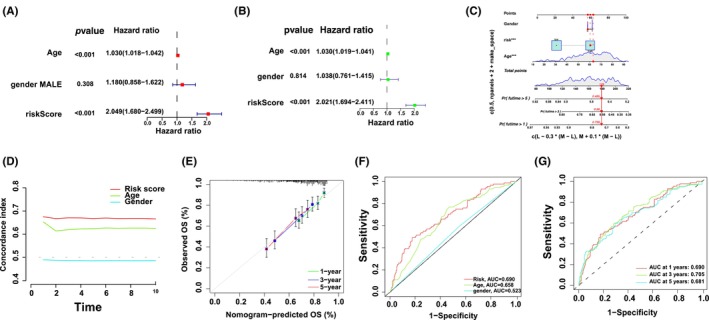
Evaluation of the prognosis risk modelling of the m6A‐associated lncRNAs and clinic characteristics in DLBCL in the entire TCGA and GEO set. (A, B) Univariable and multivariable analysis of the clinic features and risk scoring with the OS. (C) Nomograph on the foundation of sex, age and risk scoring. (D) Concordance indicators of the risk scoring and clinic features. (E) The correction plot of the nomograph forecasts the possibility of the 1‐, 3‐ and 5‐year OS. (F) ROC curves of the clinic features and risk scoring. (G) ROC curves for risk scoring within the entire TCGA and GEO set (to forecast 1‐, 3‐ and 5‐year OS).

Univariate and multivariate Cox regression analyses assessed whether risk modelling was an independent prognostic factor for DLBCL. In the multivariate Cox regression analysis, the hazard ratio (HR) of the risk scoring and 95% confidence interval (CI) were 2.04 and 1.68–2.49 (*p* < 0.001). In the univariate Cox regression analysis, the HR and 95% CI were 2.02 and 1.69–2.41, respectively, (*p* < 0.001; Figure 5A,B), indicating the potential of risk modelling as an independent prognostic factor. In contrast to clinical variables, the risk modelling of prognostic factors predominantly presented predictive utility in the nomogram analysis (Figure 5C). The consistency indicator of risk scoring and area under the receiver operating characteristic (ROC) curve (AUC) were evaluated to further assess the sensitivity and uniqueness of risk scoring for DLBCL prognosis. Over time, the consistency index of risk scoring was consistently higher than that of other clinical variables, demonstrating that risk scoring was better for DLBCL prognosis (Figure 5D). The identified and predicted 1‐, 3‐ and 5‐year OS rates showed satisfactory coherence (Figure 5E). Subsequently, time‐dependent ROC curves were used to evaluate the prognostic power of the risk class, age and sex from the TCGA and GEO databases. The AUC for the risk class was higher than that for the other factors, indicating that risk modelling based on the three m6A‐associated lncRNAs was more reliable; the AUC for risk class, age and sex predictions were 0.690, 0.658 and 0.523, respectively, in the GEO and TCGA datasets (Figure 5F). These results suggest that the prognostic factors can efficiently predict the OS of patients with DLBCL; the AUC of ROC for 1‐, 2‐ and 3‐year survival predictions was 0.690, 0.705 and 0.681, respectively, in the OS dataset (Figure 5G).

### 3.7 lncRNA ELFN1‐AS1 knockdown inhibits DLBCL cell proliferation and promotes apoptosis

**FIGURE 6:**
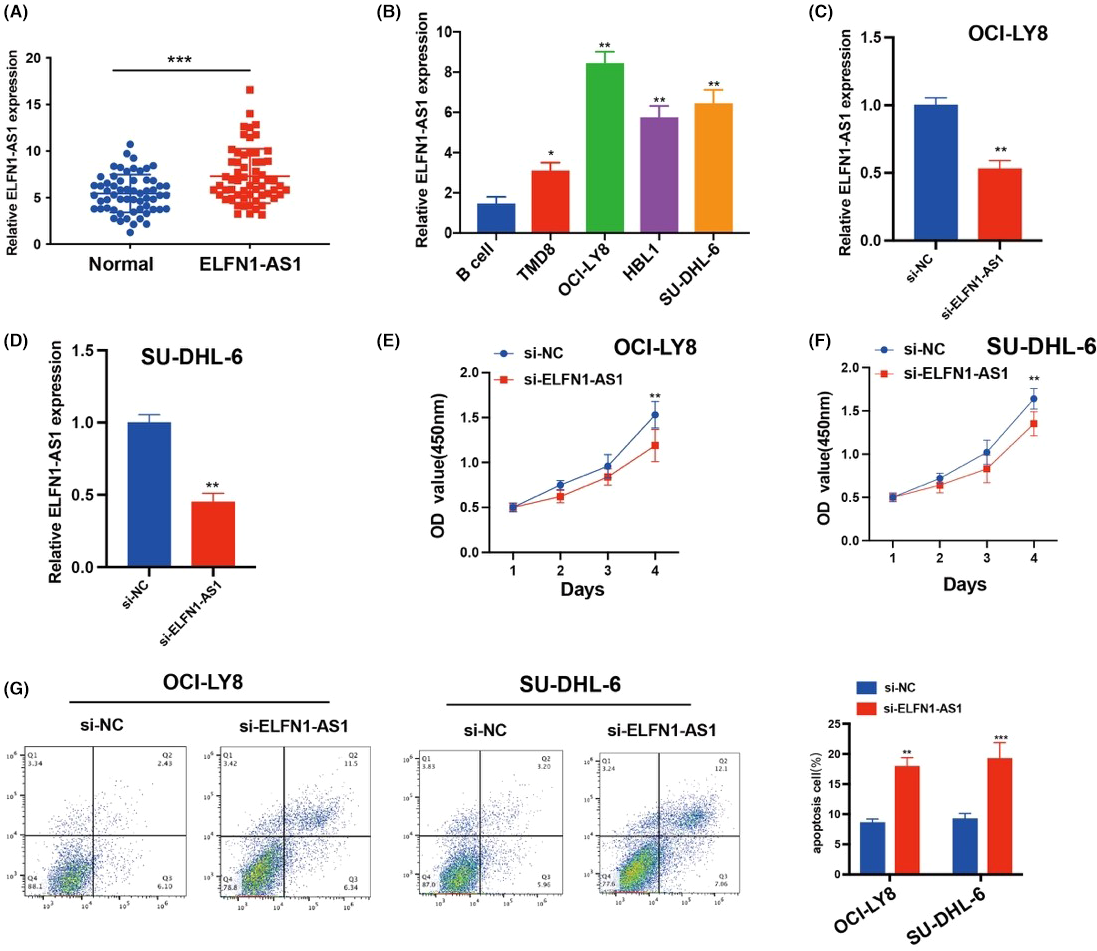
m6A‐associated lncRNAs ELFN1‐AS1 promote cell proliferation and inhibit apoptosis in DLBCL. (A) The level of ELFN1‐AS1 in the blood samples from 60 DLBCL sufferers and matched healthy controls were examined with qRT‐PCR analysis. (B) The level of ELFN1‐AS1 in B‐cell TMD8, OCl‐LY8, HBL1 and SU‐DHL‐6 cells was determined using a qRT‐PCR assay. (C, D) The efficiency of ELFN1‐AS1 knockdown was assessed using qRT‐PCR. (E, F) The proliferation of OCI‐LY8 and SU‐DHL‐6 cells was detected using CCK‐8 assays. (G) Cell apoptosis analysis of DLBCL cells with si‐ELFN1‐AS1. Data are shown as the mean ± standard deviation of three independent experiments. **p < 0.01, ***p < 0.001.

The carcinogenic effects of ELNF1‐AS1 on different cancers have been previously described. However, how ELNF1‐AS1 regulates DLBCL malignancies remains unclear. We conducted qRT‐qPCR to assess lncRNAs in the peripheral blood samples of patients with DLBCL and NC. The expression of ELFN1‐AS1 was significantly upregulated in patients with DLBCL compared with normal (Figure 6A). In DLBCL cell lines, the expression of ELFN1‐AS1 was significantly upregulated in OCI‐LY8 and SU‐DHL‐6 cells compared to that in B cells (Figure 6B), and the expression of ELFN1‐AS1 in OCI‐LY8 and SU‐DHL‐6 cells was inhibited by siRNA transfection (Figure 6C,D). The CCK‐8 proliferation assay showed that treatment with ELFN1‐AS1 siRNA decreased the proliferation of DLBCL cells (Figure 6E,F). Moreover, inhibition of ELFN1‐AS1 increased apoptosis in DLBCL cells (Figure 6G). These findings demonstrate that ELFN1‐AS1 plays a key role in stimulating DLBCL progression.

### 3.8 ELFN1‐AS1 promotes DLBCL progression through miR‐185‐5p/BCL‐2 in vitro

**FIGURE 7:**
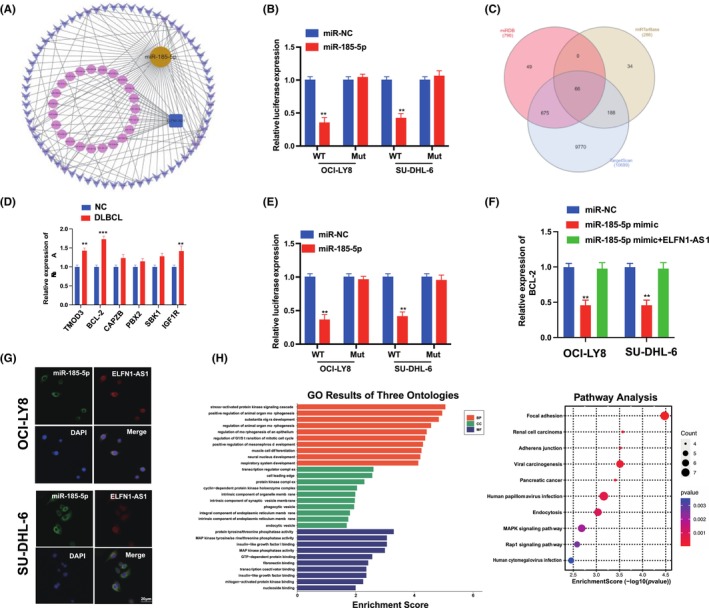
ELFN1‐AS1 promotes DLBCL progression through miR‐185‐5p/BCL‐2 in vitro. (A) ceRNA network analysis of lncRNA ELFN1‐AS1‐miRNA‐mRNA. (B) Dual‐luciferase reporter gene system assay was performed to validate the binding sites of miR‐185‐5p and ELFN1‐AS1 in DLBCL cells. (C) Network analysis of miRNA and their target genes by miRDB, miRTarBase and TargetScan. (D) The comparative levels of six mRNA candidates in DLBCL cells were detected using qRT‐qPCR. (E) Dual‐luciferase reporter gene system assay was performed to validate the binding sites of miR‐185‐5p and BCL‐2 in DLBCL cells. (F) Relative levels of BCL‐2 in DLBCL cells transfected with miR‐185‐5p mimic or miR‐185‐5p mimic bound to overexpressed ELFN1‐AS1. (G) The results of the FISH assay confirmed that ELFN1‐AS1 and miR‐185‐5P were colocalized in the cytoplasm. Scale bars are 20 μm. (H) Software R was used to analyse GO biological function and KEGG pathway enrichment. Data are shown as mean ± standard deviation of three independent experiments. **p < 0.01; ***p < 0.001.

To further explore the specific mechanism of ELFN1‐AS1 as a ceRNA in DLBCL, the target miRNAs were screened using the miRcode database (Figure 7A), in addition to 25 candidate miRNAs. Furthermore, we observed that miR‐185‐5p was expressed at low levels in various tumours.^20^, ^21^ The dual‐luciferase reporter gene system showed that miR‐185‐5p mimics significantly reduced the expression of ELFN1‐AS1 with Wt instead of Mut in OCI‐LY8 and SU‐DHL‐6 cells (Figure 7B). We predicted the possible mRNAs involved using three algorithms (miRDB, miRTarBase and TargetScan) (Figure 7C), and qRT‐PCR validation was performed for the six target genes predicted to be relatively highly expressed in DLBCL and NC, and BCL‐2 expression was significantly higher than that of matched healthy controls (Figure 7D). The dual‐luciferase reporter gene system revealed that miR‐185‐5p mimics significantly reduced the expression of BCL‐2 with Wt instead of Mut in OCI‐LY8 and SU‐DHL‐6 cells (Figure 7E). qRT‐PCR revealed that the miR‐185‐5p mimic inhibited the expression of BCL‐2; however, these alterations were reversed by ELFN1‐AS1 overexpression (Figure 7F), confirming the localization of ELFN1‐AS1 and miR‐185‐5p in the cytoplasm (Figure 7G). In addition, we used the KEGG and GO pathways to analyse the biological functions of ELFN1‐AS1 and its signalling pathways (Figure 7H). These findings demonstrated that ELFN1‐AS1 promotes DLBCL progression through miR‐185‐5p/BCL‐2 in vitro.

### 3.9 si‐ELFN1‐AS1 combined with ABT‐263 inhibits the growth of DLBCL in vitro

ABT‐263 is an orally available BAD‐like BH3 mimetic with a BCL‐2 inhibitor and is currently under clinical investigation to treat multiple cancers.^22^, ^23^ Among the 138 compounds identified, ABT‐263 had significantly different IC50 estimates across the risk groups and could be used for DLBCL therapy.

We treated OCI‐LY8 and SU‐DHL‐6 with ABT‐263; the MTT assay showed that 0.1, 1, 3 and 10 μm of ABT‐263 caused a time‐dependent, decrease in DLBCL cell growth (Figure S6A); si‐ELFN1‐AS1 combined with the ABT‐263 group significantly inhibited proliferation (Figure S6B) and promoted apoptosis in vitro (Figure S6C). Our data suggested that si‐ELFN1‐AS1 combined with ABT‐263 inhibited DLBCL cell growth and promoted apoptosis. These results revealed a synergistic therapeutic effect of si‐ELNF1‐AS1 and ABT‐263. The apoptosis‐enhancing effect of the combination therapy did not increase the killing of normal cells, highlighting the therapeutic potential of a more effective drug regimen.

### 3.10 Combretastatin si‐ELFN1‐AS1 and ABT‐263 for synergistic therapy of DLBCL in vivo

**FIGURE 8:**
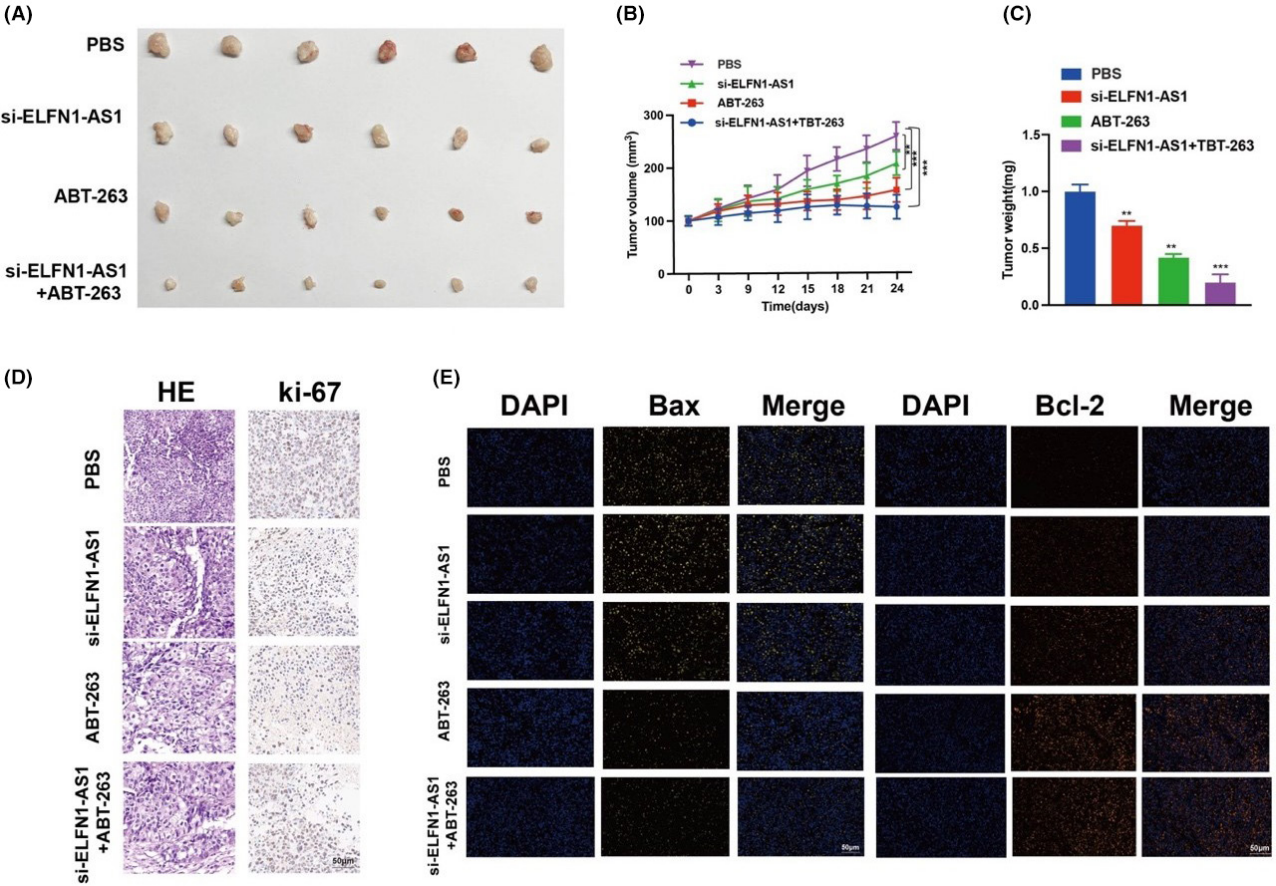
Combretastatin si‐ELFN1‐AS1 and ABT‐263 for synergistic therapy of DLBCL in vivo. (A) Representative images of combretastatin si‐ELFN1‐AS1 and ABT‐263 of the tumours. (B, C) Effects of suppression of combretastatin si‐ELFN1‐AS1 and ABT‐263 on tumour volume and tumour weight. (D) HE and Ki‐67 were detected by immunohistochemistry. (E) Bax (yellow) and BCL‐2 (orange) were detected using immunofluorescence on mouse tumour tissue. Scale bar: 50 μm. Data are shown as mean ± standard deviation of three independent experiments. **p < 0.01, ***p < 0.001.

We examined the role of si‐ELFN1‐AS1 and ABT‐263 in combination therapy in vivo and observed that tumour volume and weight were significantly lower in the si‐ELFN1‐AS1 combined with ABT‐263 group (Figure 8A–C). Ki‐67 immunofluorescent staining was used to identify cellular proliferative activity. As shown in Figure 8D, there were fewer Ki‐67 in the si‐ELFN1‐AS1 combined with the ABT‐263 group than in the other groups. Haematoxylin and eosin staining showed more necrotic areas in the si‐ELFN1‐AS1 combined with the ABT‐263 group than in the other groups.

Meanwhile, the weak fluorescence of Bax (yellow) and strong fluorescence of BCL‐2 (orange) (Figure 8E) in tumour tissues in the si‐ELFN1‐AS1 combined with the ABT‐263 group were in contrast to those in the other groups. These findings indicated a strong suppressive role of si‐ELFN1‐AS1 combined with ABT‐263 in the proliferation of DLBCL cells in vivo.

## 4 DISCUSSION

Recent studies have indicated that m6A mRNA methylation promotes the differentiation of haematopoietic stem cells and progenitor cells.^24^, ^25^, ^26^ Nevertheless, the effects of m6A on DLBCL tumourigenesis remain poorly understood; therefore, researchers are increasingly focusing on determining ncRNA hallmarks that can predict survival and response to immunotherapy in DLBCL sufferers.^27^, ^28^ Several studies have shown that m6A modifications may exert a modulatory effect on tumour pathogenesis.^29^, ^30^ m6A modulators can modify specific lncRNAs to aid the persistence of malignancy‐related m6A and lncRNAs in various cancer types. m6A modifications of lncRNAs have been observed to influence tumourigenesis and progression,^30^, ^31^ and lncRNAs may target m6A modulators that act as competing endogenous RNAs to promote cancer invasion and development. These findings suggest that m6A modifications target lncRNAs, and the interaction between the functions of lncRNAs and m6A modifications needs to be explored further to determine prognostic biomarkers or therapeutic targets for tumours.^32^, ^33^, ^34^

Here, the prognostic utility of 293 m6A‐associated lncRNAs was investigated using TCGA, GTEx and GEO databases. TCGA and GEO corroborated the prognostic ability of 25 m6A‐associated lncRNAs; three were utilized to establish m6A‐associated lncRNA models that could predict OS in patients with DLBCL. Subsequently, patients with DLBCL were divided into low‐ and high‐risk groups according to the mid‐value of the risk scoring, with the high‐risk group presenting with significantly worse clinical outcomes. Multivariate Cox regression analysis revealed that the m6A‐associated lncRNA model was associated with OS risk factors. Receiver operating characteristic (ROC) analyses demonstrated that the model‐based method outperformed traditional methods based on clinical features in predicting the survival of patients with DLBCL. Our team created a nomograph displaying full agreement between the identified and predicted OS rates at 1, 3 and 5 years, and the observed and predicted OS rates at 1, 3 and 5 years showed good agreement. Our risk modelling based on three m6A‐associated lncRNAs independently linked to OS exhibited remarkable accuracy, and this predictive modelling approach can be used to identify novel markers in future studies.^35^ TMB is the sum of somatic cell‐encoded variants associated with the occurrence of new antigens that trigger antitumor immune activity.^36^ Recent studies have shown that TMB is an effective marker for predicting response to PD‐L1 therapy.^37^, ^38^, ^39^ No differences were observed in TMB between the low‐ and high‐risk groups. Hence, we infer that such a predictive modelling method may offer a dependable immune response‐related biomarker for cancer treatment. Additionally, this study offers novel insights into the molecular‐level causal links between m6A‐associated lncRNAs and DLBCL.

In clinical practice, pathological staging is an independent factor that affects DLBCL prognosis.^40^ Patients with same‐stage DLBCL consistently exhibit diverse clinical results, indicating the inaccuracy of the current staging system in predicting patient survival and treatment outcomes and highlighting the inhomogeneity of DLBCL. Therefore, additional prognostic and therapeutic markers need to be identified. The m6A‐associated lncRNA model established herein offers a novel approach to DLBCL prognosis. These findings offer insight into the processes and mechanisms of m6A‐modified lncRNAs.^41^, ^42^

In our study, a novel network of m6A‐associated lncRNAs ELFN1‐AS1/miR‐185‐5p/BCL‐2 in DLBCL was constructed using biological tools. Functional experiments revealed that the proliferation of DLBCL cells was inhibited following the interference of ELFN1‐AS1. BCL2 is a well‐known regulator gene that inhibits apoptosis by contributing to the intrinsic apoptosis pathway.^43^, ^44^ A previous study revealed that the miR‐185‐5p/BCL‐2 regulatory axis plays a vital role in the prognosis of breast cancer. Next, we revealed the regulatory mechanisms of ELFN1‐AS1, miR‐185‐5p and BCL‐2 in DLBCL cells and discovered that ELFN1‐AS1 upregulated BCL‐2 by sponging miR‐185‐5p, which could be a key mechanism and therapeutic target for DLBCL treatment.

ABT‐263 (Navitoclax) is a BH3 mimetic drug targeting anti‐apoptotic B‐cell lymphoma‐2 (BCL‐2) family proteins,^45^ with potential anticancer activity against various types of cancer. Clinical phase 1 and phase 2 studies have shown that it is safe for patients with lung cancer, has few side effects and has significant efficacy.^46^, ^47^ This potential is due to its high binding affinity for anti‐apoptotic proteins of the Bcl‐2 family, which disrupts the sequestration of pro‐apoptotic proteins. Extending our in vitro findings, we observed that ABT‐263 combined with si‐ELFN1‐AS1 suppressed viability and induced apoptosis in OCI‐LY8 and SU‐DHL‐6 cells, and in vivo studies showed that after Si‐ELNF1‐AS1 combined with ABT‐263, the expression of Bax decreased. The expression of BCL‐2 increased in tumour tissues stained by immunofluorescence. The tumour volume and weight decreased. These mechanistic results confirm the success of the combined antitumor therapy. In addition, the number of tumour cells decreased significantly, and the number of undesirable tissues increased significantly after the combination of drugs; thus, the combination of BH3‐mimetics and gene therapy is a promising approach that should be evaluated in further clinical studies.

In conclusion, our study offers insights into DLBCL prognosis and may facilitate elucidation of the causal link between m6A and the regulation of lncRNAs. This predictive model exhibited promising reliability in identifying patients with DLBCL who may respond well to immunotherapy. In addition, our study revealed a synergistic effect of si‐ELNF1‐AS1 and ABT‐263. The apoptosis‐enhancing and tumour‐inhibiting effects of combination therapy highlight the therapeutic potential of a more effective component of the drug regimen.

This study is the first to explore the potential correlation between m6A‐related genes and the DLBCL immune microenvironment, suggesting that the tumour immune microenvironment may influence m6A modifications in DLBCL. However, this study has several limitations. First, all data were extracted from online databases, and data from biochemical experiments were unavailable for validation. Second, although this study showed a potential correlation between m6A‐related genes and the immune microenvironment of DLBCL, there is a limited understanding of the relationship between m6A methylation regulators and ELFN1‐AS1. Future plans include conducting in‐depth studies on the mechanisms underlying the sensitivity, evasion and resistance of lncRNAs to m6A methylation modifications.

## AUTHOR CONTRIBUTIONS

**Ashuai Du:** Data curation (equal); writing – original draft (equal). **Yingxue Lu:** Conceptualization (equal); writing – original draft (equal). **Qinglong Yang:** Data curation (equal); writing – original draft (equal).

## FUNDING INFORMATION

Guizhou Provincial People's Hospital Youth fund project (GZSYQN202205). Traditional Chinese medicine project in Guizhou Province (QZYY‐2023‐014).

## CONFLICT OF INTEREST STATEMENT

The authors declare that they have no competing interests.

## Supporting information


Figures S1–S6

## Data Availability

The data supporting the findings of this research are available from the corresponding author upon request.
