# Supplementary material for: m6A‐related lncRNAs as potential biomarkers and the lncRNA ELFN1‐AS1/miR‐182‐5p/BCL‐2 regulatory axis in diffuse large B‐cell lymphoma
Source: J Cell Mol Med. 2023 Dec 1;28(2):e18046. doi: 10.1111/jcmm.18046 (PMC10826449; doi:10.1111/jcmm.18046)
Supplement: Supplementary file 1 — Figures S1–S6 [file JCMM-28-e18046-s001.docx]

**SUPPLEMENTARY FIGURE**


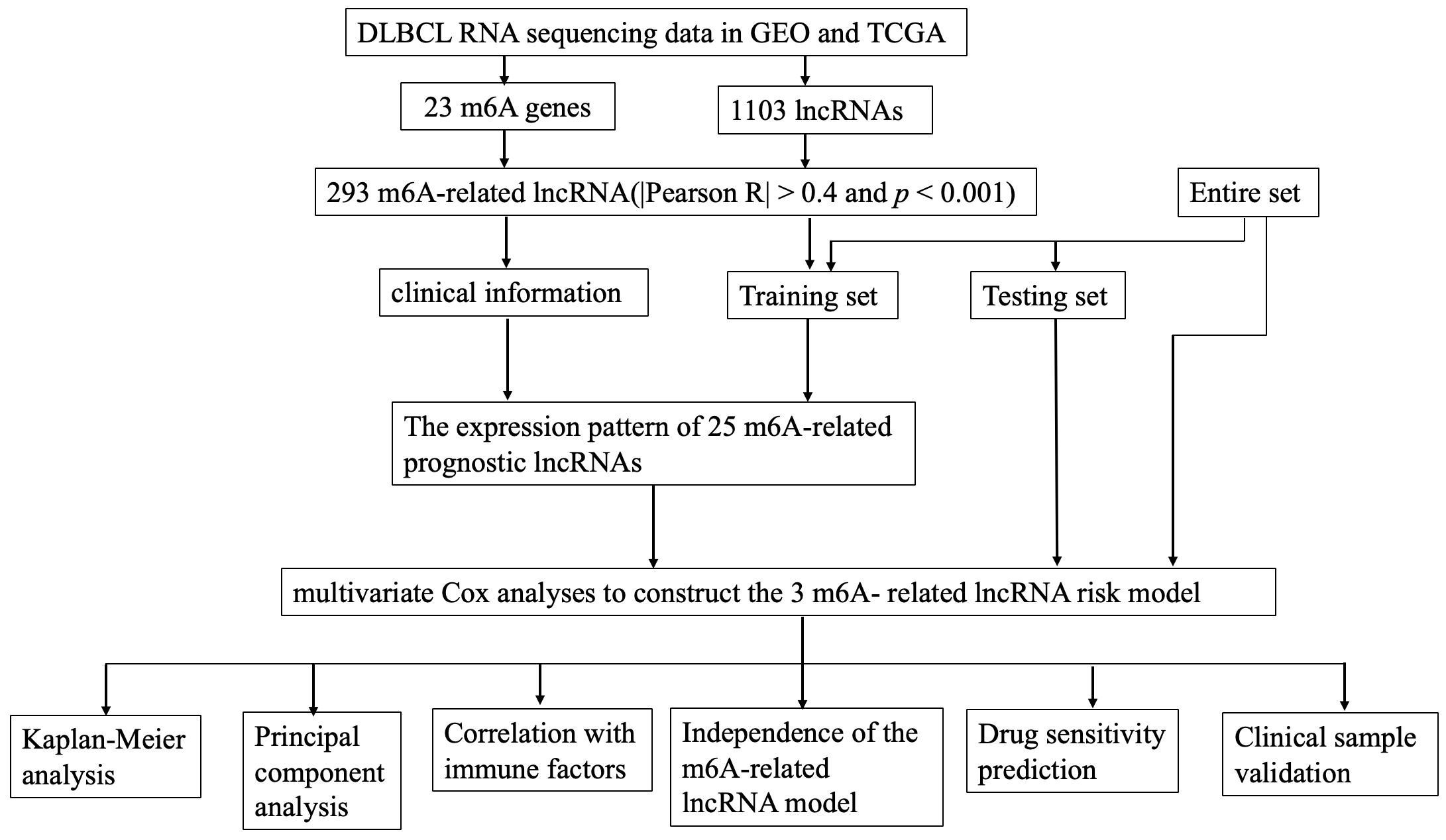


**Supplementary Figure 1** Flow chart of the current investigation.

**Supplementary Figure 2 Risk modeling for DLBCL sufferers on the foundation of m6A-associated lncRNAs**

(A) Univariable Cox regressive analyses revealed that the chosen lncRNAs were remarkably related to clinical prognoses. (B) The adjustment parameters (log λ) of OS-associated proteins were chosen to realize the cross-verification of the deviation curve. As per the minimum standard and 1-se standard, vertical imaginary lines were plotted at the optimum value. (C) The LASSO coefficient profile of 25 OS-associated lncRNAs and a vertical imaginary line were plotted at the value selected by 10-fold cross-verification.

**Supplementary Figure 3 K-M curves of OS diversities** **layered by** **sex and age between the low-risk and high-risk groups in the entire TCGA and GEO set**

(A-B) K-M curves of OS diversities layered by age (≤65 or >65).

(C-D) K-M curves of OS diversities layered by sex.

**Supplementary Figure 4 PCA between the low-risk group and high-risk group on the foundation of (A) Entire genetic expression profiles, (B) 23 m6A genes, (C) 293 m6A-associated lncRNAs, and (D) risk modeling on the foundation of the representative profiles of the three m6A-associated lncRNAs within the entire TCGA and GEO set.**


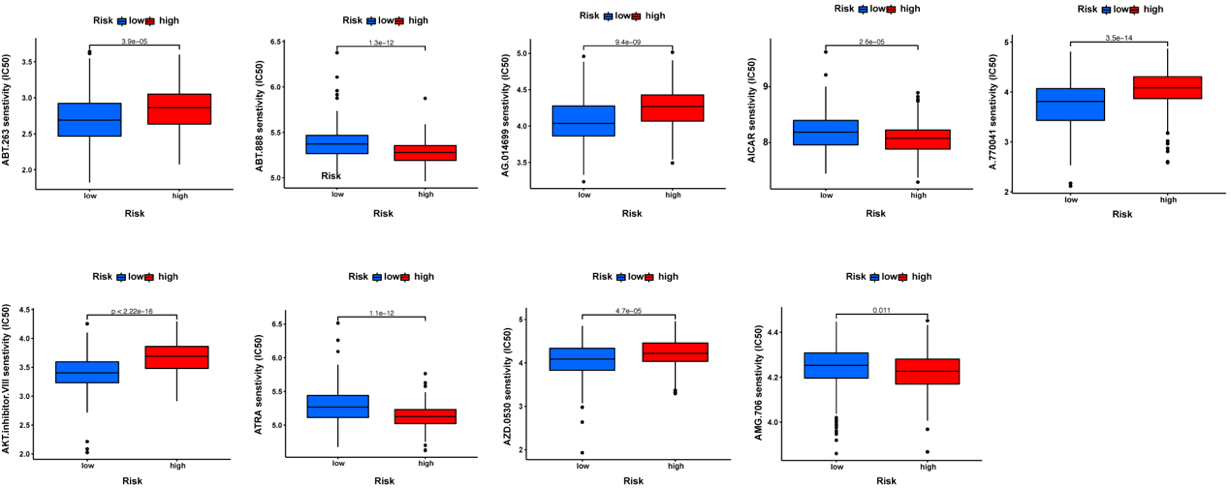


**Supplementary Figure 5 Determination of new promising compounds that target the m6A-associated lncRNA model**

**
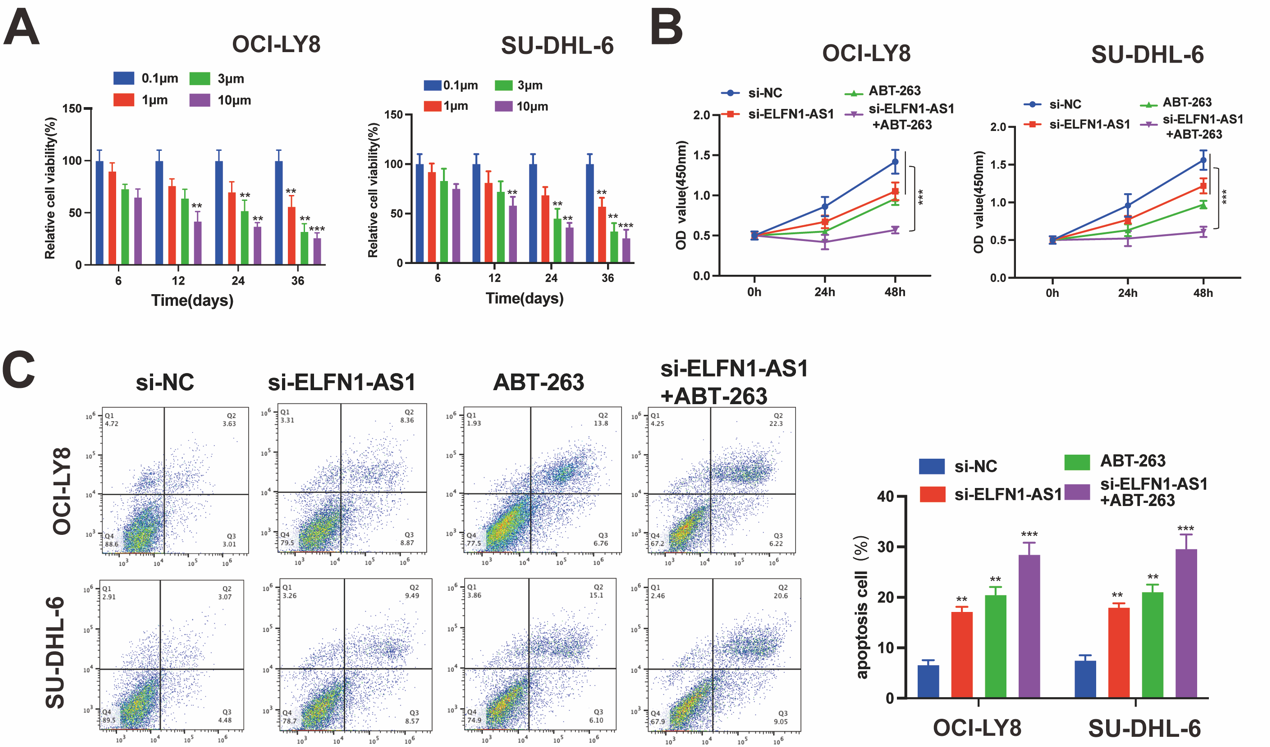
**

**Supplementary Figure 6 Knockdown of ELFN1-AS1 combined with ABT-263 inhibits DLBCL cell growth and proliferation and promotes apoptosis *in vitro***

(A) CCK-8 assay analysis of cell viability after treatment with different doses of ABT-263. (B) The proliferation of OCI-LY8 and SU-DHL-6 cells was detected. (C) Flow cytometry was employed to identify the cell apoptosis of OCI-LY8 and SU-DHL-6 cells after 24 h. Data are shown as mean ± standard deviation of three independent experiments. ***P* *<*0.01, ****p* <0.001.
